# Evaluating Characteristics of *De Novo* Assembly Software on 454 Transcriptome Data: A Simulation Approach

**DOI:** 10.1371/journal.pone.0031410

**Published:** 2012-02-27

**Authors:** Marvin Mundry, Erich Bornberg-Bauer, Michael Sammeth, Philine G. D. Feulner

**Affiliations:** 1 Evolutionary Bioinformatics, Institute for Evolution and Biodiversity, Westfaelische-Wilhelms-University, Muenster, Germany; 2 Functional Bioinformatics, Centre Nacional d'Anàlisi Genòmica (CNAG), Barcelona, Spain; University of Hyderabad, India

## Abstract

**Background:**

The quantity of transcriptome data is rapidly increasing for non-model organisms. As sequencing technology advances, focus shifts towards solving bioinformatic challenges, of which sequence read assembly is the first task. Recent studies have compared the performance of different software to establish a best practice for transcriptome assembly. Here, we adapted a simulation approach to evaluate specific features of assembly programs on 454 data. The novelty of our study is that the simulation allows us to calculate a model assembly as reference point for comparison.

**Findings:**

The simulation approach allows us to compare basic metrics of assemblies computed by different software applications (CAP3, MIRA, Newbler, and Oases) to a known optimal solution. We found MIRA and CAP3 are conservative in merging reads. This resulted in comparably high number of short contigs. In contrast, Newbler more readily merged reads into longer contigs, while Oases produced the overall shortest assembly. Due to the simulation approach, reads could be traced back to their correct placement within the transcriptome. Together with mapping reads onto the assembled contigs, we were able to evaluate ambiguity in the assemblies. This analysis further supported the conservative nature of MIRA and CAP3, which resulted in low proportions of chimeric contigs, but high redundancy. Newbler produced less redundancy, but the proportion of chimeric contigs was higher.

**Conclusion:**

Our evaluation of four assemblers suggested that MIRA and Newbler slightly outperformed the other programs, while showing contrasting characteristics. Oases did not perform very well on the 454 reads. Our evaluation indicated that the software was either conservative (MIRA) or liberal (Newbler) about merging reads into contigs. This suggested that in choosing an assembly program researchers should carefully consider their follow up analysis and consequences of the chosen approach to gain an assembly.

## Introduction

454 transcriptome sequencing is widely used as a cost effective sequencing method, especially for non-model organisms [1]–[31]. Concentrating the sequencing effort on the expressed part of the genome not only saves costs, it allows analysis of the expressed part of the genome, which is not easily predicted from the genome sequence alone. Splice patterns, versatile combinations of exons, can be identified, and gene expression rates can be estimated and compared. In addition, single nucleotide polymorphisms (SNPs) and simple sequence repeats (SSRs) within the coding part of the genome can be determined.

Most analyses that utilise transcriptome data require assembled reads. With next generation sequencing (NGS), DNA molecules are fragmented, size-selected, amplified, and high-throughput sequenced resulting in reads of a length which is specific for the respective NGS technology. This fragmentation procedure is reversed *in silico* by merging overlapping reads into contigs during the assembly process. The study presented here focuses on the performance of software for *de novo* assembly of cDNA reads generated by 454 sequencing. In studies lacking a sequenced genome, it is not possible to assemble the reads by mapping them onto a reference genome. Instead all reads have to be aligned against each other, i.e. *de novo* assembled. Despite the higher costs compared to other NGS technologies, 454 is still widely used because of the long reads it produces, facilitating read alignment during the *de novo* assembly. Other sequencing technologies, such as Illumina, are constantly increasing their read length and supersede 454 especially in terms of throughput and per base pair costs. In addition, new technologies being developed for example the semiconductor technology of Ion Torrent. Therefore, the assembly of around 200 bp long reads, as evaluated in the study presented here, likely will persist as a bioinformatics challenge.

For the *de novo* assembly of 454 transcriptomic reads the following assemblers are most widely used: CAP3 [32] (TGICL [33], wrapper for CAP3), MIRA [34] (est2assembly [35], wrapper for MIRA), Newbler [36], Seqman NGen© , CLC bio©, and the web application EGassembler [37] (see Table 1). Not all of these assemblers are specifically intended for transcriptome data. In contrast to a genome consisting of few long continuous stretches (linkage groups or chromosomes), the transcriptome is comprised of many transcripts that are variable in length. The complexity of assembling a transcriptome is further exacerbated by varying expression levels, resulting in an uneven distribution of reads amongst the diverse transcripts. Even if experimental cDNA normalization aims to reduce the dynamic range of expression it usually does not result in an even distribution of transcripts [8]. In addition, alternative splicing results in multiple isoforms, which share partial sequence information [38].

**Table 1:** Assembler software recently used for *de novo* assembly of 454 transcriptome data.§

Assembler	Organism
**CAP3 [31]**	*Amaranthus tuberculatus* [2]; *Conyza canadensis* [3]; *Momordica charantia* [4]; *Oncopeltus fasciatus* [5]; *Oryza longistaminata* [6]; *Papaver somniferum* * [7]; *Pisum sativum* * [8]; *Pteridium aquilinum* [9]; *Trichostrongylus colubriformis* [10];
**MIRA [33]**	*Anguilla anguilla* [11]; *Bathymodiolus azoricus* [12]; *Cochliomyia hominivorax* [13]; *Cucurbita pepo* [14]; *Fagopyrum esculentum* and *F. tataricum* [15]; *Pisum sativum* [8]; *Pteridium aquilinum* [9]; *Schmidtea mediterranea* [16]; *Thamnophis elegans* [17]; *Trialeurodes vaporariorum* * [18];
**Newbler [35]**	*Agrilus planipennis* [19]; *Cajanus cajan* [20]; *Cimex lectularius* [21]; *Euphausia superba* [22]; *Oncopeltus fasciatus* [5]; *Paulinella chromatophora* [23]; *Phytoseiulus persimilis* [24]; *Teladorsagia circumcincta* [25]; *Thamnophis elegans* [17]; *Vigna radiata* [26];
**Seqman NGen **©	*Crotalus adamanteus* [27]; *Littorina saxatilis* [28]; *Oncorhynchus mykiss* [29];
**CLC bio ©**	*Coregonus clupeaformis* [30]; *Tigriopus californicus* [31];
**EGassembler [36]**	*Amaranthus tuberculatus* [2]; *Conyza canadensis* [3];

*Utilising a wrapper TGICL [33] or est2assmbly [35].

§ For more studies refer to Table 1 in [1].

These intrinsic features of the transcriptome pose special challenges for any assembly software. A recent study by Kumar and Blaxter compared transcriptome assemblers by analysing 454 cDNA reads from *Litomosoides sigmodontis*, a nematode, and evaluated the resulting contigs [1]. The quality of read assemblies were assessed for basic assembly metrics, such as various measurements of bases used, contig number, and length. In addition, contigs were compared with previously existing sequence databases. Besides presenting a very comprehensive evaluation of different software solutions, some aspects have not been addressed exhaustively: (1) The analysis of basic assembly metrics usually suffers from the fact that optimal values are not known when only using real data. Although it may seem tempting to simply assume that longer contigs represent a better assembly, this might not necessarily be the case, e.g. if reads of different transcripts are concatenated. (2) The comparison of assemblies with pre-existing sets of reference sequences from other organisms might be misleading. The best performing assembler does not necessarily always match well with reference sequences, even when these references originate from the same species because the transcriptome varies depending on tissue, time point, and abiotic factors [39]–[42]. (3) Due to some sequence similarity between transcripts, reads originating from different transcripts can be merged into one contig during the assembly process. Without knowledge of the origin of reads, it is difficult to determine the extend to which an assembler produces chimeric contigs, i.e. contigs containing reads from different transcripts.

We used a novel approach to assess the performance of assembler software. By applying a simulation approach we circumvent some of the problems mentioned above. Given a transcriptome, the simulator carried out *in silico* gene expression, reverse transcription, fragmentation and 454 sequencing. In contrast to real 454 reads, the exact origin of each simulated read was known. Utilising this information it was possible to merge reads with a minimum of one base pair overlap, independent of sequence information. This way, we knew an ideal solution (Model Assembly MA), which was assigning all reads to their original transcripts while merging reads as efficiently as possible with the given amounts of data (one single 454 plate). Therefore the MA was the optimal solution of the assembly problem given the data. The same simulated reads were assembled using assembly software which operated on sequence information only. The resulting assemblies could be compared to the MA. The MA provided reference values for basic assembly metrics, such as contig count and contig length. Additionally, the MA could be used as a reference data set against which to compare the output contigs of the assemblers to determine specificity and sensitivity measurements. Assessing the amount of reads aligning back to multiple contigs identified alignment ambiguity and redundancy in the assemblies. As we knew from which transcript each simulated read originates, it was, in addition, possible to identify reads of different origin joined to form one chimeric contig and quantify the extent of chimera formation in the different assemblies.

In our study we created simulated reads based on a description of the human transcriptome (GRCh37.58). The human data set was chosen due to the comprehensive amount of data available and the complexity and size of the transcriptome. In addition, we used real 454 reads from a human tissue pool in order to compare the simulation approach with a realistic experimental setup. The assemblers tested in this study are CAP3 [32], MIRA [34], Newbler [36], and Oases [43]. These assemblers had been chosen as they are frequently used in non-model organism transcriptome studies and are freely distributed stand-alone applications (see Table 1). Although Oases is primarily designed for shorter Illumina reads, it was included in this study because it is specifically designed for transcriptome data.

## Results

### The simulated and real data set

**Figure 1:**
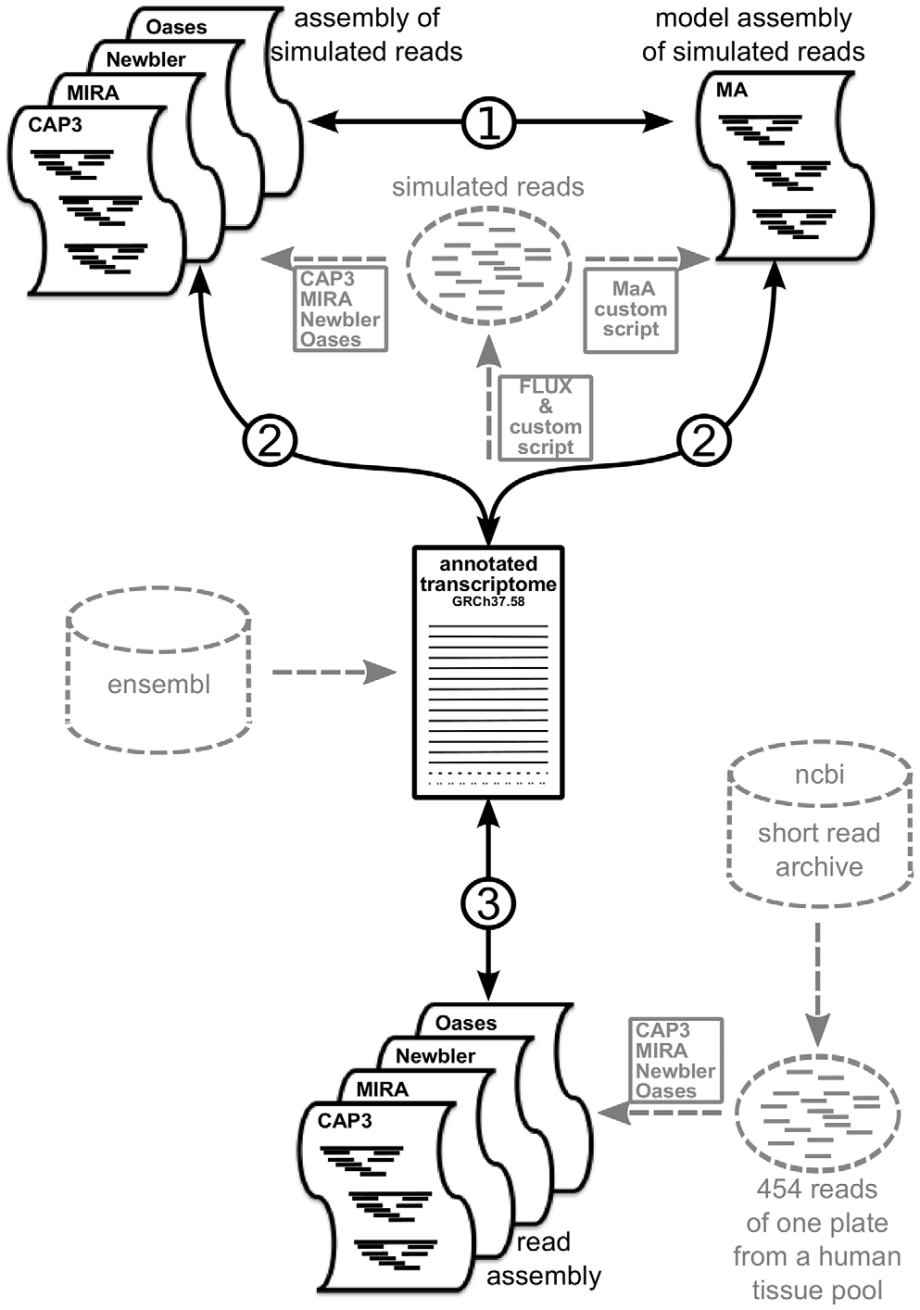
Workflow and comparison scheme for assembler evaluation. Workflows are shown in grey, comparisons between data sets in black. To evaluate the performance of different assemblers three comparisons were performed: 1) Different assemblies of simulated reads were compared to a Model Assembly (MA), which was based on positional information. 2) Different assemblies of simulated reads were compared to a transcriptome annotation. The MA was compared in the same way to provide reference values for the evaluated measurements. 3) Different assemblies of real reads were compared to the transcriptome annotation to compare the simulation approach to values from a real data set.

The simulation produced 3′340′245 cDNA fragments (for details see Material and Methods). We randomly discarded all but 800'000 fragments to match the amount of a “typical” single 454 sequencing run. Gene expression, reverse transcription, fragmentation, and 454 sequencing was simulated based on a human transcriptome annotation [44] in the following manner: the cell profiles are randomly assigned according to a modified Zipf's law as observed universally in RNA expression interrogations [45]. Subsequently, in silico expressed transcript molecules have been subjected to reverse transcription to recast 5′ to 3′-representation biases in libraries that are reversely transcribed before fragmentation [46]. Then simulated fragmentation was carried out employing a mechanical model of molecule breakage [47]. Fragments obtained were sub-sampled in the sequencing process, additionally mimicking errors typical for the sequencing chemistry [36], [47]. The resulting simulated reads (800'000 reads of a mean read length ∼220 bp) were assembled using four different *de novo* transcriptome assembly programs, namely CAP3, MIRA, Newbler, and Oases. For comparison a “real” 454 data set (NCBI Short Read Archive Accession: SRX002932) containing 823'575 sequences (454 FLX reads with an average length of 250 bp) was obtained and assembled using the same assembly programs. For the simulation approach we generated a Model Assembly (MA) based on the origin of each read. In the MA, reads were merged into contigs using position information if they overlapped by at least a single base pair. Figure 1 illustrates the workflow (light grey) with details on the data sets and comparisons (black) made to evaluate the assemblers. We utilised the MA ultimately as a reference point for the evaluation of transcriptome assemblers (Comparison 1 in Figure 1). The simulated reads were created from the transcriptome annotation, and after assembly, compared back to it (Comparison 2 in Figure 1). Finally, real reads from an independent experiment were also assembled and compared to the transcriptome annotation (Comparison 3 in Figure 1).

### Basic assembly metrics

To allow comparisons between the assemblies of different assembly programs (run under default parameters for transcriptome assembly; details are given in Material and Methods), singletons and contigs shorter than 100 bp were discarded before subsequent analysis. Standard metrics describing the assembly, such as number of contigs, total bases used in the assembly, number of large contigs (>1 kbp), number of base pairs used in large contigs, maximal, average, and median contig length, and N50 value, were used to compare the assembly programs. The N50 value is defined as the contig length where half the assembly is represented by contigs of this size or longer. We included N50 values for comparison with other studies even if it is not strictly applicable for transcriptome assemblies [1].

**Figure 2:**
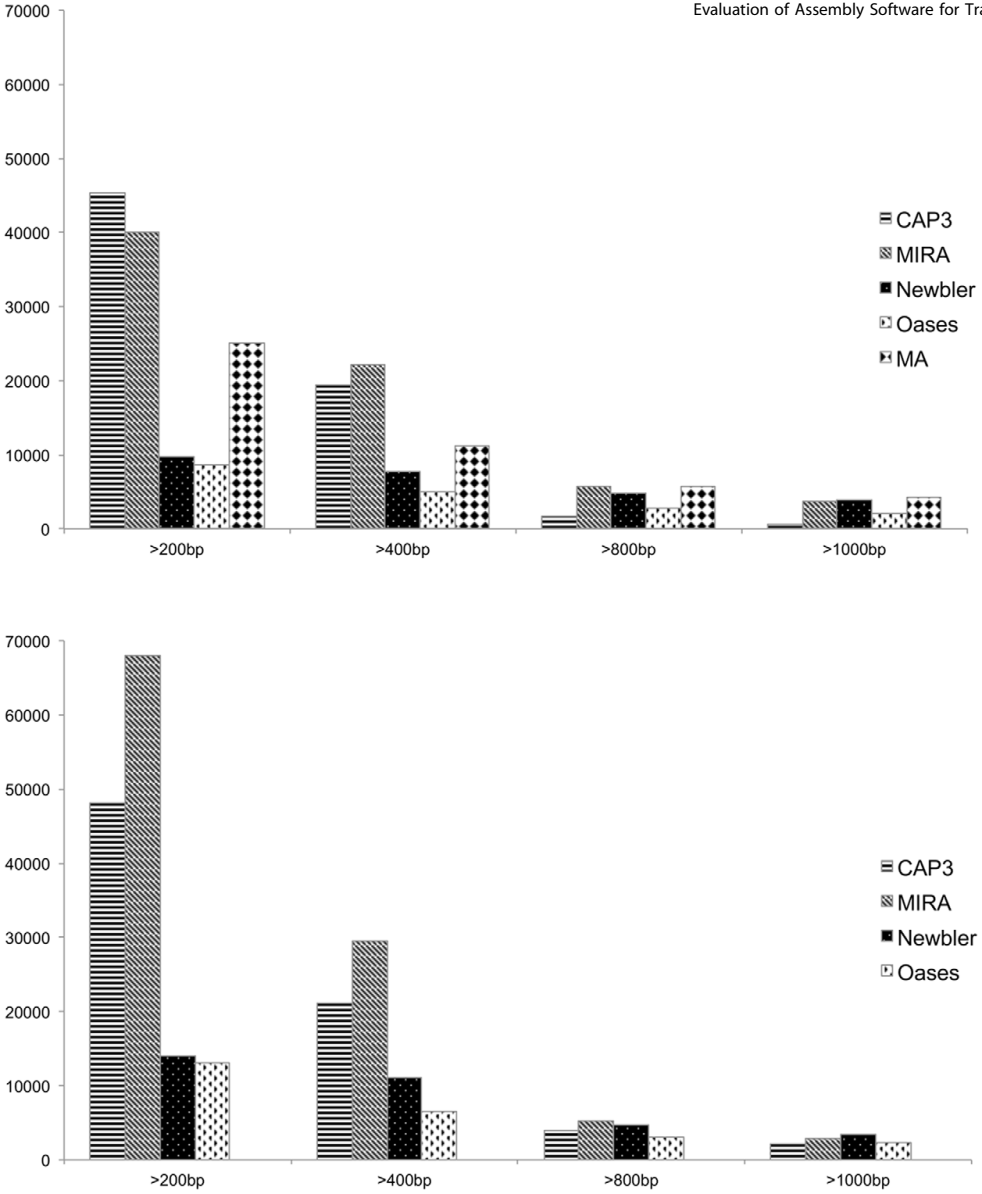
Cumulative contig lengths for different assemblies. Counts of contigs longer than 200, 400, 800, and 1000 base pairs for the different assemblies. Assemblies of simulated (top) and real 454 reads (bottom) are shown in separate diagrams.

The number of contigs produced by the algorithmically similar programs MIRA and CAP3 is about 4 times higher than the amount of contigs produced by Newbler and the algorithmically very different Oases. This was observed in the assemblies of the simulated data (Table 2) as well as of the real reads (Table 3). Newbler and Oases produced less contigs than were present in the MA, while MIRA and CAP3 produced more. Accordingly, comparing the amount of bases output into contigs by the assembly programs, MIRA and CAP3 assemblies added up about twice the number of base pairs compared to Newbler and Oases. This held for both simulated and real data sets. The number of contig bases output by CAP3 and MIRA was above but close to the amount of bases in the MA. Oases put out less than half the bases of the MA. Newbler produced on average longer contigs than other assemblers tested in this study (highest mean and median contig length), even though this did not amount to overall more base pairs. The mean and median contig length retrieved with MIRA, CAP3, and Oases were quite similar to the values of the MA. Figure 2 shows the distribution of contig lengths. CAP3 produced many short contigs and few long contigs. MIRA and both versions of Newbler produced more long contigs than CAP3, but MIRA also output a high amount of short contigs. CAP3 and Oases produced fewer long contigs, while MIRA and Newbler constructed almost as many long contigs as present in the MA. Newbler and Oases assemblies held fewer short contigs compared to the MA. Overall, the contig length comparison showed similar results for simulated and real read assemblies. Besides the current version of Newbler (2.3) we also tested a prerelease version (2.5p1 beta version as far as applicable, for details see Material and Methods), which performed very similar in all the analysis (results not shown). Wall-clock run times for each program varied between minutes to around a day on a 2.6 GHz AMD Opteron 2435 server with 16 GB RAM. At this magnitude the runtime is of less importance than the quality of an assembly, but might become relevant if one wants to explore the parameter space or analyse larger or multiple data sets [1].

**Table 2:** Basic assembly metrics (simulated 454 reads).

	CAP3	MIRA	Newbler	Oases	MA
Number of contigs*	45'422	40'129	9'774	11'355	24'993
Total bases*	19'147'862	22'855'498	12'764'265	7'937'884	18'152'459
Number of contigs (> = 1 kbp)	606	3'683	3'938	2'138	4'337
Total bases (in contigs > = 1 kbp)	779'806	6'626'729	9'614'255	4'686'216	9'935'980
Max contig length	13'981	17'958	17'915	17'906	17'958
Mean contig length	421	569	1'305	699	726
Median contig length	376	427	797	331	330
N50	425	602	2'128	1'351	1'214
Time taken	341 min	859 min	34 min	10 min**	N/A

* Only contigs >100 bp.

** Summed time for velveth, velvetg, and Oases.

**Table 3:** Basic assembly metrics (real 454 reads).

	CAP3	MIRA	Newbler	Oases
Number of contigs*	50'381	76'126	14'633	16'862
Total bases	22'062'745	31'495'153	11'728'579	9'020'336
Number of contigs (> = 1 kbp)	2'106	2'964	3'365	2'261
Total bases (in contigs > = 1 kbp)	2'963'339	4'188'919	6'007'896	3'890'312
Max contig length	4'859	3'958	8'611	8'461
Mean contig length	437	413	801	534
Median contig length	364	337	565	300
N50	458	456	1'025	837
Time taken	1731 min	816 min	790 min	8 min**

*Only contigs >100 bp.

**Summed time for velveth, velvetg, and Oases.

### Assembly evaluation

Assemblers could also be evaluated on how well the respective assembly recaptures already known sequences. We compared the assemblies to the human transcriptome annotation from Ensembl. We aligned the assembled contigs to the transcriptome and vice versa, evaluating specificity and sensitivity of the comparison. Specificity was defined as the relative amount of contigs covering at least 80% of a respective transcript in the Ensembl annotation (BLAST e-value<10^−9^ for details see Material and Methods). Specificity was high for all assemblies of the simulated reads, which were directly created out of the Ensembl transcriptome (91 to 95%; Table 4). Assuming that the Ensembl annotations were comprehensive, contigs not matching the transcriptome were either too short to cover at least 80% of a respective transcript or were potential misassembled contigs. The assemblies of real reads, which stem from RNA of different human cell lines, showed a lower and broader range of specificity (49 to 79%; Table 5). Amongst the real read assemblies Newbler and Oases scored highest, while MIRA produced the lowest amount of contigs that were contained in the transcriptome. Sensitivity was defined as the relative amount of Ensembl transcripts contained in the assemblies (for details see Material and Methods). For the assemblies of the simulated reads the sensitivity showed a broader range (2 to 13%; Table 4). This indicated performance differences between the assemblers. Newbler and MIRA were most sensitive, while CAP3 was least sensitive. The real read assemblies all showed a low sensitivity (6 to 8%; Table 5).

**Table 4:** Comparison between simulated 454 read assemblies and transcriptome.

	CAP3	MIRA	Newbler	Oases	MA
Specificity absolute	42'697/45'422	37'587/40'129	8'932/9'774	10'398/11'355	23'737/24'993
Specificity relative	94.00%	93.67%	91.39%	91.57%	94.97%
Sensitivity absolute	3'140/146'962	14'920/146'962	18'723/146'962	9'379/146'962	23'985/146'962
Sensitivity relative	2.14%	10.15%	12.74%	6.38%	16.32%

**Table 5:** Comparison between real 454 read assemblies and transcriptome.

	CAP3	MIRA	Newbler	Oases
Specificity absolute	30'256/50'381	37'376/76'126	11'505/14'633	12'065/16'862
Specificity relative	60.05%	49.10%	78.62%	71.55%
Sensitivity absolute	10'487/146'962	11'209/146'962	11'857/146'962	9'543/146'962
Sensitivity relative	7.14%	7.63%	8.07%	6.49%

Ultimately we aimed to evaluate assembler performance based on how well their assembly of the simulated reads approximates the MA (Table 6). All assemblers achieved a rather high specificity: 91 to 95% of the contigs generated by the assemblers were present in the MA. The CAP3 and MIRA assemblies had the highest specificity, while Newbler showed the lowest specificity. Sensitivity was lower than specificity: 15 to 41% of the MA contigs were found in the simulated read assemblies. The MA had the highest contig overlap with the MIRA assembly (41%) followed by Newbler (34%), CAP3 (17%), and Oases (15%). Specificity and sensitivity indicated that MIRA produced the assembly that was most similar to the MA (Table 6).

**Table 6:** Comparison between simulated 454 read assemblies and model assembly.

	CAP3	MIRA	Newbler	Oases
Specificity absolute	43'312/45'422	37'930/40'129	8'856/9'774	10'582/11'355
Specificity relative	95.35%	94.52%	90.61%	93.19%
Sensitivity absolute	4'202/24'993	10'329/24'993	8'530/24'993	3'671/24'993
Sensitivity relative	16.81%	41.33%	34.13%	14.69%

Ambiguity within the assemblies was evaluated by aligning simulated reads back to assembled contigs (Table 7). The greatest majority of reads could be mapped back to MA contigs. For the MA, we knew that only reads of a common transcript had been assembled. Nevertheless, while mapping the reads to the contigs, some reads aligned to multiple contigs due to sequence similarity between transcripts. This is showing the intrinsic redundancy within the data set. A similar high proportion of aligned reads as for the MA could only be found in the MIRA contigs. For the other assemblies some reads did not find a good match in the assembled contigs. Redundancy in the assembly was revealed by reads mapping back to multiple contigs. CAP3 and MIRA had by far the most reads with multiple hits, whereas Newbler and Oases had fewer reads mapped to multiple contigs than the MA. Another form of ambiguity, chimeric contigs arose when reads, which originated from different transcripts, were assembled into the same contig. These chimeras might cause major artefacts in analysis following a transcriptome assembly, like detection of sequence or expression variation. Our simulation approach kept track of the origin of every read and allowed us to find and quantify the amount of misplaced reads forming chimeric contigs in the assemblies. Table 8 shows that MIRA and CAP3 have a high proportion of non-chimeric contigs (86% and 85%, respectively), whereas Newbler produced only 62% of non-chimeric contigs. As the Oases software did not allow tracing reads during the assembly process we could not determine with certainty which reads contributed to the contigs and therefore could not evaluate the extend of non-chimeric contigs directly. Transcriptome assemblies are especially challenging since genes with multiple transcripts are difficult to distinguish using sequence information only. Therefore, we expected these genes to be particularly prone to misalignments. Out of the 800'000 reads simulated 64'630 originate from non-alternative spliced genes. The average proportions of misplaced reads for contigs representing genes with and without alternative splicing are presented in Table 8. As expected, genes with a single transcript isoform showed a lower average proportion of misplaced reads per contig in all assemblies. This indicates, that no assembler performed particularly well in assembling genes with multiple splicing variants, reflecting the specific challenges of assembling transcriptome data. But again MIRA and CAP3 outperformed Newbler in this aspect.

**Table 7:** Alignment ambiguity between simulated reads and assembled contigs.

	CAP3	MIRA	Newbler	Oases	MA
Contigs hit	45'410/45'422	40'108/40'129	9'771/9'774	11'342/11'355	24'983/24'993
Reads mapped (out of 800'000)	708'344	786'490	709'680	689'079	798'768
Reads mapped to multiple contigs	609'429	611'832	202'681	223'616	294'738

**Table 8:** Evaluation of chimera formation.

	CAP3	MIRA	Newbler
Non chimeric contigs absolute	38'429/45'422	34'558/40'129	6'138/9'957
Non chimeric contigs relative	85%	86%	62%
Average proportion of misplaced reads AS	5.27%	4.61%	11.70%
Average proportion of misplaced reads non-AS	0.88%	0.91%	2.82%

AS: Genes with alternative splicing.

Non-AS: Genes without alternative splicing.

## Discussion

### The Model Assembly as a reference for comparing assembler performance

Previous studies that evaluated assemblers for *de novo* transcriptome data, compared the assembly of different programs against previously determined EST sequences for the target species and transcriptome data of related organisms [1]. In this study we adopted a simulation approach to evaluate a given assembly and compare different assemblies. Thus, we benefited from knowing the optimal solution for the assembly problem given the data. We created a Model Assembly (MA) with a minimal overlap of one bp ensuring that no assembler outperforms the MA, therefore the MA could be used as the gold standard. First, the MA provided reference values for the diverse metrics, on which the assembler were assessed and compared to each other. Second, we evaluated how close the different assemblies match the MA. And finally, this approach allowed us to evaluate chimeric contigs in the assembly directly. As the simulation may not have captured all confounding processes involved in real experiments, e.g. PCR read chimeras, we also evaluated a comparable real data set. In assemblies of real experimental data the optimal solution was not known. Nevertheless, in the case presented in this study, the human transcriptome annotation was most likely a very good proxy. Altogether, the combination of simulated and real experimental data provided further insights on general advantages and shortcomings of different software solutions for 454 *de novo* transcriptome assembly.

### Comparing the assembly metrics

The interpretation of assembly quality based on metrics like contig length was difficult without reference values. For example, longer contigs are not always good indicators of assembly quality; if an assembler simply concatenates all reads, the result would be an assembly with a high median contig length although it is a large chimeric sequence. The MA provided reference values for basic assembly measures so these values could be assessed accordingly. We exploited this feature to reveal Newbler produced contigs with a higher median length, average length, and N50 length, than the MA. This suggested that Newbler was merging reads into contigs that originate from different transcripts. This assumption was further strengthened by the lower number of base pairs output in the Newbler assembly compared to the MA. The higher amount of chimeric contigs and the higher average amount of misplaced reads per contig in the Newbler assembly relative to the other assemblies (Table 8), which was only possible to evaluate directly due to our simulation approach, further confirmed these conclusions. MIRA and CAP3 seemed to be more conservative in merging reads, resulting in lower median contig length but higher numbers of base pairs used in the assemblies. A large total number of bases used in the assembly pointed towards some degree of redundancy in the MIRA and CAP3 assemblies. Again our simulation approach allows us to directly show the higher redundancy in these assemblies (Table 7). Oases produced contigs of a similar length as CAP3, MIRA, and MA, but output a low number of bases in the assembly suggesting that the software discarded some read information. Comparing assembly metrics for the simulated and real data set (see Table 2 and 3), led to similar conclusions, revealing major differences between Newbler on the one side and MIRA plus CAP3 on the other side.

### Specificity and sensitivity of the transcriptome assemblies

As expected, the MA had the highest specificity and sensitivity (Table 3). In the simulation, 800'000 reads were produced representing the amount of sequences generated by one 454 sequencing run. Due to the amount of data used for the assembly, sensitivity was rather low (16%) and a specificity of 95% indicated that not all contigs in the MA represent complete transcripts, i.e. some transcripts were not completely covered by reads. These results confirmed that a single 454 sequencing run did not allow for a complete restoration of a whole (human) transcriptome, resulting in low sensitivity scores and incomplete specificity. However we could compare specificity and sensitivity across different software solutions to determine performance differences. We evaluated a prerelease (2.5p1) version of Newbler along with the current 2.3 version, as Kumar and Blaxter [1] showed major performance differences between versions. We only found minor differences in the performance between the two Newbler versions in any of the metrics or comparisons we evaluated (results not shown). These findings were in the line with results by Ewen-Campen et al. [5].

The specificity scores of the different assemblies for simulated reads were all very high. This might be expected as the reads have been directly generated from the transcriptome, resulting in almost every contig mapping back to the reference. For the real data set, specificity differed more between the assemblies (Table 5), and indicated that Newbler was most successful in restoring complete length transcripts. MIRA showed a low specificity, which might be caused by the conservative merging of reads. Essentially, MIRA escaped producing chimeric contigs but as a consequence failed to produce long contigs and generates redundancy in the assembly. The sensitivity scores showed slight variations due to the low amount of initial reads. For both simulated and real read sets, Newbler and MIRA were the most sensitive assemblers. The relatively low specificity, together with the low number of contigs and amount of bases used in the Oases assembly indicated that Oases might not be the right choice for transcriptome assembly of 454 reads. This might not be surprising as this software was designed for shorter reads and not for ∼200 bp reads as used in this study. The Oases assembly might be improved by using a multiple k-mer strategy, but for the scope of this study we decided to evaluate software with their default settings.

### Conclusions

As we used a simulation approach we were able to identify general features of different software for *de novo* 454 transcriptome assembly. In summary, our analysis indicated that Newbler performed best in restoring full-length transcripts at the cost of a higher proportion of chimeric contigs. In contrast, MIRA was particularly conservative in combining reads. This resulted in more fragmented transcripts and a certain degree of redundancy in the assembly. Depending on the analysis following the assembly, researchers might favour different features of assemblers. Downstream variation detection might suffer substantially from chimeric reads, which produce false positive variation calls. Therefore one might prefer a conservative approach as performed by MIRA. Other studies interested in the expressed sequences might prefer an optimal restoration of full-length transcripts with minimal redundancy. Here, Newbler might be the better choice for assembly, despite some degree of chimeric contigs. All assessed approaches can be assumed to benefit from experimental improvement, like e.g. normalization of expression levels, or tuning parameter settings specific for the data analysed, but the overall tendencies of characteristic differences between approaches we describe here are less expected to change. Furthermore we focus here on the *de novo* assembly of a specific human transcriptome. The assembly problem might vary depending on tissue type, expression profile, and species under consideration. Nevertheless we outline in our study how a simulation approach can guide decision on assembly strategy and support the choice of parameters. Simulations on reads obtained under similar experimental conditions in related species can also provide valuable information for the design and the analysis of RNA-Seq experiments in species with an *a priori* unknown transcriptome composition.

## Materials and Methods

### Data sets

For the qualitative evaluation of sequence assemblers, we simulated 454 ESTs *in silico*. On the basis of the human genome and transcriptome annotation (Ensembl [44] GRCh37.58) the FluxSimulator [48] (v20090831) simulated gene expression (20000 Cells, 200 Million Molecules), reverse transcription (transcription start site variation: 25, poly-A shape and scale: 0, random primers) and fragmentation (physical, lambda 900, cDNA cut-off 500–800 bp; parameters not mentioned were left at default values). The human transcriptome was chosen as a start point for the simulation due to its quality especially with respect to the knowledge about different isoforms of genes. A custom python script (available on request from the authors) resembling the approach of MetaSim [49] simulated the 454 sequencing process with 100 flow cycles. Throughout all these processes the information about which transcript a fragment/read originated from was maintained. This allowed creation of a Model Assembly (MA). The MA was not based on overlapping sequence information between reads but instead was based on the knowledge of the origin of each read. Reads were merged into contigs when they shared a common origin and overlapped by at least one base pair. Any assembler operating on sequence information could not produce a better assembly than the MA. For comparison we repeated the analysis with a real 454-FLX sequenced human transcripts [50]. The sequenced transcripts originated from the microarray quality control A sample (NCBI Short Read Archive Accession: SRX002932). It consisted of pooled RNA from different cell lines and therefore should give a good representation of the human transcriptome.

### Assemblers

We compared the performance of following assemblers: CAP3 (version for Linux with an Intel processor) [32], MIRA (3.2.0rc3) [34], Newbler (2.3 and 2.5p1) [36], and Oases (0.1.18) [43]. Based on the algorithms the assembly software uses, the assemblers can be grouped into two different classes. Overlap-Layout-Consensus based assemblers (MIRA, CAP3, Newbler) are usually employed in the assembly of longer reads such as those produced by 454 sequencing. De Bruijn graph assemblers (Oases) are primarily designed for short read data, e.g. from Illumina sequencing [51]. Although we only studied long-read data sets we nevertheless evaluated the performance of Oases. We did so as Oases is explicitly designed for the assembly of transcripts. All assemblers were run under default parameters with the following required adjustments for 454 transcriptome data: MIRA: denovo,est,accurate,454 -GE:not = 4; Newbler2.3,Newbler2.5: cDNAMode = True, numCPU = 4; Oases: k = 31. The prerelease version of Newbler 2.5 (2.5p1) used in this study contained a bug (known to the developers) that causes the software to fail on reading in certain cDNA reads in fasta format. In order to assess the software, we had to eliminate reads crashing the program manually. For the simulated reads, Newbler 2.5 was run with only 793'430 reads instead of 800'000 reads. On the real read data set, we were not able to run the software at all. After assembly, contigs less than 100 bp in length and singletons (singletons could not be determined in the Oases assembly) were discarded for subsequent analysis (this was done to ensure comparability between the output of the different assemblers as some keep while other discard singletons and/or contigs shorter 100 bp).

### Comparative evaluation of assemblers

We compared the total number of bases in an assembled contigs, the amount of contigs longer 200 bp, 400 bp, 800 bp, and 1 kbp, mean, median, maximum, N50 contig length (the smallest contig size in which half the assembly is represented), and run times of all evaluated assemblers for both the simulated and the real data set. As Oases uses a preliminary assembly produced by velvet (specifically, the applications, velveth and velvetg) we summed run times over all steps. These statistics were collected to determine which assembler approximates the MA best.

In addition to the simulation approach, we compared the assemblies of real and simulated data to the transcriptome. We expected that the assemblers would perform comparably on the simulated and real data. We calculated the following optimality criteria to validate our simulation approach and evaluate the performance of the different assemblers: (1) Specificity: This measure described the relative amount of contigs in the assembler's output which were also contained in the transcriptome or MA. We considered a contig to be present in the transcriptome or MA if it had a BLAST [52] hit with an e-value<10^−9^ and the hit covered at least 80% of the length of the transcriptome or MA sequence. (2) Sensitivity: The relative amount of transcriptome sequences or MA contigs, which were contained in the output of one assembler (BLAST) and covering at least 80% of the length of the output contig. Figure 1 illustrates performed comparisons between the different data sets. (3) Ambiguity: Aligning simulated reads back to assembled contigs, we evaluated how many reads map to multiple contigs (multiple BLAST hits above the e-value threshold of 10^−9^) to assess redundancy in the assembled contigs. Besides, we evaluated the origin of reads joined into the same contig. Non-chimeric contigs aligned only reads of the same transcript origin. For each contig, we determined the proportion of misplaced reads (reads mapped to contigs originating from different transcripts - chimeras). We calculated the average proportion of misplaced reads over all contigs for alternatively spliced and non-alternatively spliced genes, separately.
